# *N* ^6^‐methyladenine profiling of low‐input multiplex clinical samples on transcriptome reveals RNA modifications implicated in type 2 diabetes and acute myocardial infarction

**DOI:** 10.1002/ctm2.1165

**Published:** 2023-01-11

**Authors:** Shanshan Qin, Kun Yang, Shaoqing Han, Yushu Yuan, Jing Mo, Mengyao Xiao, Yin Yang, Yafen Wang, Xin Fang, Fang Wang, Wei Zhang, Song‐Mei Liu, Xiaocheng Weng, Xiang Zhou

**Affiliations:** ^1^ College of Chemistry and Molecular Sciences Key Laboratory of Biomedical Polymers of Ministry of Education Wuhan University Wuhan China; ^2^ Department of Clinical Laboratory Center for Gene Diagnosis and Program of Clinical Laboratory Zhongnan Hospital of Wuhan University Wuhan China; ^3^ School of Pharmaceutical Sciences Wuhan University Wuhan China; ^4^ Department of Preventive Medicine Simpson Querrey Institute for Epigenetics Northwestern University Feinberg School of Medicine Chicago Illinois USA

Dear Editor,

RNA *N* ^6^‐methyladenines (m^6^A) not only play an essential role in normal biological processes but also participate in the pathogenesis of diseases including type 2 diabetes (T2D) and comorbidities.^1^, ^2^ However, conventional technologies (e.g. m^6^A‐seq)^3^ require large amounts of input materials often prohibitive for clinically‐feasible biospecimens (e.g. a few millilitres of blood or tissue from fine needle aspiration), we, therefore, developed a Strategy of Low‐Input Multi‐barcode m^6^A‐seq (SLIM‐m^6^A‐seq) to facilitate transcriptome‐wide m^6^A profiling with limited RNA. Using this new technology, we implicated novel m^6^A modifications and pathways in the development of acute myocardial infarction (AMI) in patients with T2D.

Technically, SLIM‐m^6^A‐seq exploited the barcoding strategy to encode multiple samples with unique barcodes before pooling, library construction, and sequencing (Figure 1A and Table S1).^4^, ^5^ The resulting profiles can be split by barcodes (Table S2) and traced back to the original samples. We first tested the feasibility of SLIM‐m^6^A‐seq in HeLa cells and calibrated them with spike‐in oligonucleotides (Note S1 and Figure S1). Then we compared SLIM‐m^6^A‐seq of a pooled sample containing barcoded mRNA varying from 200 to 10 ng with m^6^A‐seq of 800 ng mRNA (Note S1). The SLIM‐m^6^A‐seq results from input levels of 200 and 100 ng showed similar distribution (e.g. chromosome 1 in Figure S2A) and complexity with 2.56% relative standard deviation (RSD), compared to the data from m^6^A‐seq in 800 ng input, albeit substantially different starting amounts (Figure S3A).

**FIGURE 1:**
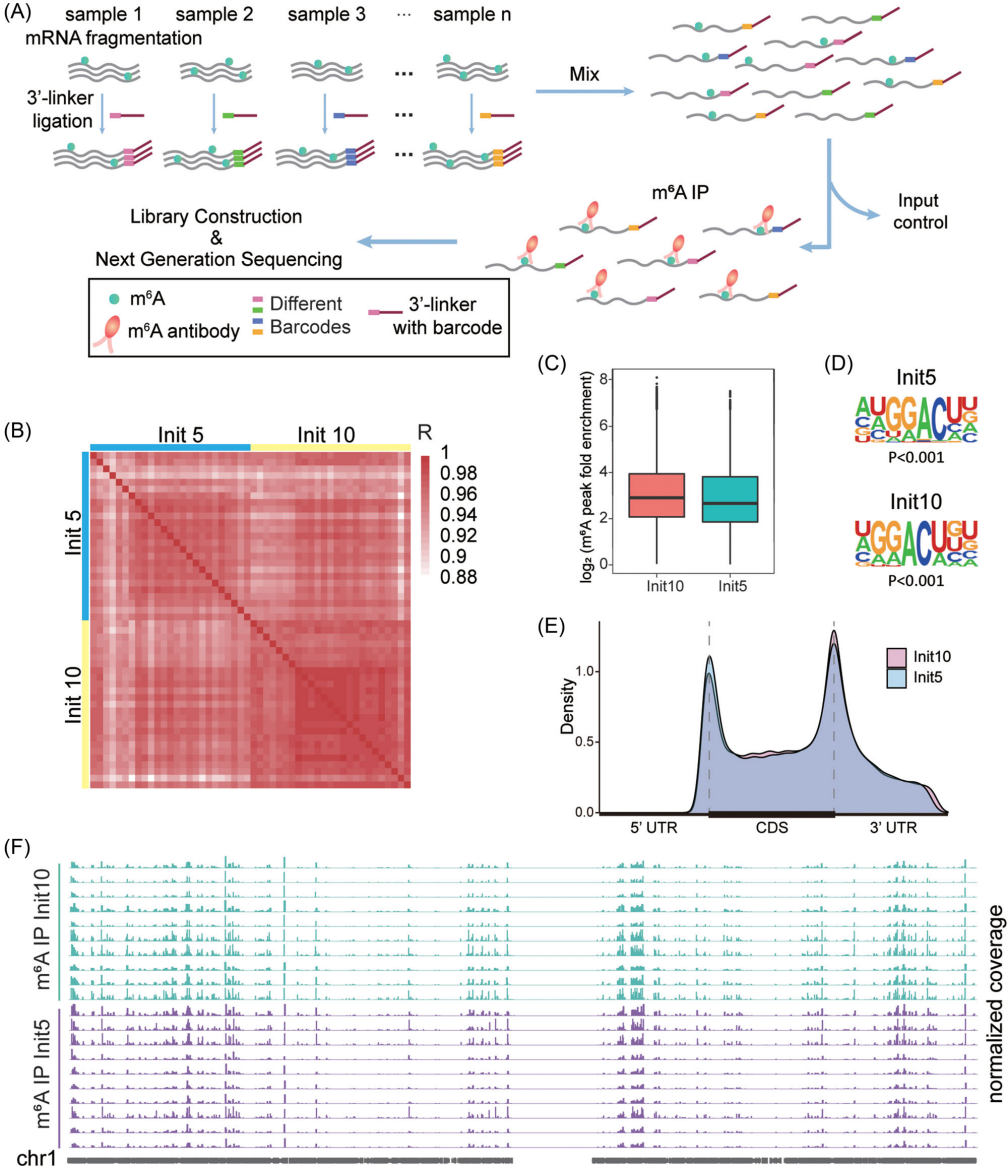
Technical design and validation of Strategy of Low‐Input Multi‐barcode m^6^A‐seq (SLIM‐m^6^A‐seq) in low‐input samples. (A) The schematic diagram shows major technical steps of SLIM‐m^6^A‐seq that accommodate multiple samples by exploiting a barcoding strategy. (B) The hierarchical clustering shows a universal correlation of m^6^A profiles across individually barcoded samples (Init5, 5 ng each; Init10, 10 ng each). (C) The box plot shows comparable m^6^A fold enrichments of Init5 and Init10 samples. (D) The sequence motifs of the detected m^6^A enriched regions in Init5 and Init10 samples (*p* < 0.001) are consistent with the RRACH motif. (E) The density plots show consistent distribution patterns of m^6^A peaks in Init5 and Init10 samples. UTR: untranslated region; CDS: coding sequence. (F) Chromosome 1 is shown as an example of consistent m^6^A profiles in the Init5 and Init10 sets of samples (10 random samples each)

We further validated the capability of SLIM‐m^6^A‐seq for low‐input samples in two 25‐sample sets containing 10 ng (Init10) or 5 ng (Init5) fragmented mRNA from HEK293T cells. Overall, greater than 90% mapping rates on transcriptome were achieved for these samples (Table S3). A similar number of m^6^A peaks was detected, with an RSD of 34.26% in Init10 and an RSD of 37.43% in Init5 (Figure S4A), as well as a consistent number of m^6^A‐containing genes across different barcodes (Figure S4B). The m^6^A profiles across the two sets were highly correlated (Figure 1B and Figure S4C,D), thus confirming technical reproducibility in low‐input samples. The barcoded samples in Init5 or Init10 sets exhibited high reproducibility of complexity with RSD ≦ 20% in both sets with or without m^6^A immunoprecipitation (IP), although the complexity would be reduced with smaller input as expected (Figure S4E). The enrichment folds of m^6^A in individually barcoded samples were comparable between both sets – 6.22‐folds in Init5 and 7.51‐folds in Init10 (Figure 1C and Figure S5A). The RRACH motifs (Figure 1D and Figure S5B) and consistent m^6^A distributions (Figure 1E) were discovered across all samples, as shown in an example of chromosome 1 (Figure 1F).

We then profiled a cohort of 20 blood samples (Figure S6 and Table S4) from patients with T2D (*n* = 10) and T2D with AMI (T2D+AMI) (*n* = 10) matched for age, sex and blood pressure to demonstrate the clinical feasibility of SLIM‐m^6^A‐seq. A similar complexity was observed in all samples with the ratio of distinct/total reads ranging from 0.84 to 0.90 and an RSD of 1.74% (Figure 2A). The principal component analysis suggested transcriptome‐wide differences between these two groups (Figure 2B). In total, 3223 dysregulated genes (|log_2_ (fold change)| ≥ 1, adjusted *p* < 0.05) were detected between these two groups (Figure 2C), with 1845 up‐regulated and 1378 down‐regulated in T2D+AMI (Figure 2D). A panel of 14 differential genes was further established through the leave‐one‐out cross‐validation and the elastic net regularization (Figure 2E and Table S5). Note that, 7234 m^6^A peaks were found with typical distributions (Figure 2F) and RRACH motifs (Figure 2G). SLIM‐m^6^A‐seq revealed an elevated overall m^6^A modification level in T2D+AMI patients (Figure 2H), which was confirmed by high‐performance liquid chromatography‐tandem mass spectrometry (Figure 2I and Table S6) and also reflected by the fact that 806 genes with increased and 260 genes with reduced m^6^A modification levels in T2D+AMI patients (Figure 2J).

**FIGURE 2:**
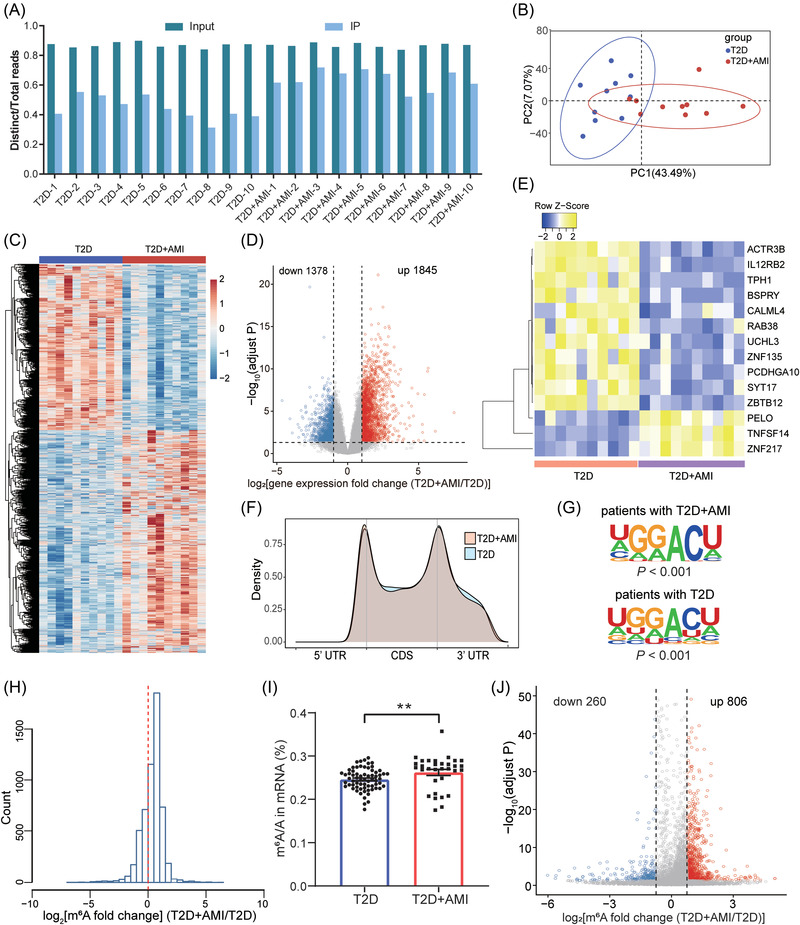
Implementation of Strategy of Low‐Input Multi‐barcode m^6^A‐seq (SLIM‐m^6^A‐seq) in human blood samples from patients with type 2 diabetes (T2D) and T2D + acute myocardial infarction (AMI). (A) Sequencing complexity is shown by the ratio of distinct reads to total reads in T2D and T2D+AMI samples. (B) The Principle Component Analysis (PCA) shows the general clustering of the transcriptomes by diagnosis classification: T2D and T2D+AMI. (C) Hierarchical clustering using the Z‐scores of differential genes between T2D and T2D+AMI identified using SLIM‐m^6^A‐seq. (D) The magnitude of upregulated or down‐regulated genes in T2D+AMI patients compared to T2D patients is shown in the volcano plot. (E) The heatmap shows the most important differential genes selected using the elastic net regularization and retained after the leave‐one‐out cross‐validation (LOOCV). (F) The density plot shows consistent distribution patterns of m^6^A peaks across samples from patients with T2D and T2D+AMI. UTR: untranslated region; CDS: coding sequence. (G) The sequence motifs of the m^6^A peaks detected by SLIM‐m^6^A‐seq (*p* < 0.001) are consistent with the RRACH motif. (H) The histogram shows a globally higher m^6^A modification level in patients with T2D+AMI, relative to patients with T2D. (I) The ratios of m^6^A/A (%) of mRNA quantified by high‐performance liquid chromatography‐tandem mass spectrometry (HPLC‐MS/MS) are shown. Bars represent mean ± standard deviation (SD) in patients with T2D or T2D+AMI (**p < 0.01). (J) The volcano plot shows m^6^A enriched genes in T2D+AMI samples and T2D samples. Those genes with increased m^6^A abundance (up) are highlighted in red, while those genes with decreased m^6^A abundance (down) are shown in blue (adjusted *p* < 0.05)

Notably, the expression level of the m^6^A demethylase *FTO* decreased evidently in T2D+AMI (Figure 3A), supported by real‐time quantitative polymerase chain reaction (RT‐qPCR) (Figure 3B and Tables S6 and S7). The conjoint analysis uncovered 392 genes exhibiting different combinations of expression and m^6^A modification patterns (|log_2_ (fold change)| ≥ 1, adjusted *p* < 0.05 for expression change, and ≥ 1.68‐fold enrichment for m^6^A). Of these intersecting genes, 311 genes exhibited concordant direction of changes, and 81 showed contrary alterations (Figure 3C). Gene ontology (GO) analysis of the 392 overlapping genes revealed that the biological processes related to T2D+AMI were involved in protein kinase activities, inflammatory response, apoptosis, and angiogenesis (Figure 3D),^6^ which have been implicated in vascular biology, inflammation, and myocardial injury (Table S5).^7^ Of the 392 intersecting differential genes, several inflammation‐ or atherosclerosis‐related genes and angiogenesis activators showed correlation (Pearson |R| ≥ 0.5) with one or more clinical cardiac and inflammatory biomarkers (Figure 3E). Of note, *TNFSF14*, a credible upregulated gene explored in our differential gene analysis and relevant to T2D and angiocardiopathy,^8^, ^9^ showed reduced m^6^A in T2D+AMI. This observation indicated that the effect of m^6^A in the progression of AMI during T2D could be through regulating the expression of disease‐related genes. The GO analysis was further supported by the observed changes in clinical indexes relevant to myocardial injury and inflammation between T2D+AMI and T2D (Figure 3F). Together with mediators in apoptosis and angiogenesis, these differential m^6^A‐containing genes were relevant to inflammation and cardiomyocyte death,^10^ suggesting the significant role of m^6^A in the occurrence of T2D+AMI through these mechanisms.

**FIGURE 3:**
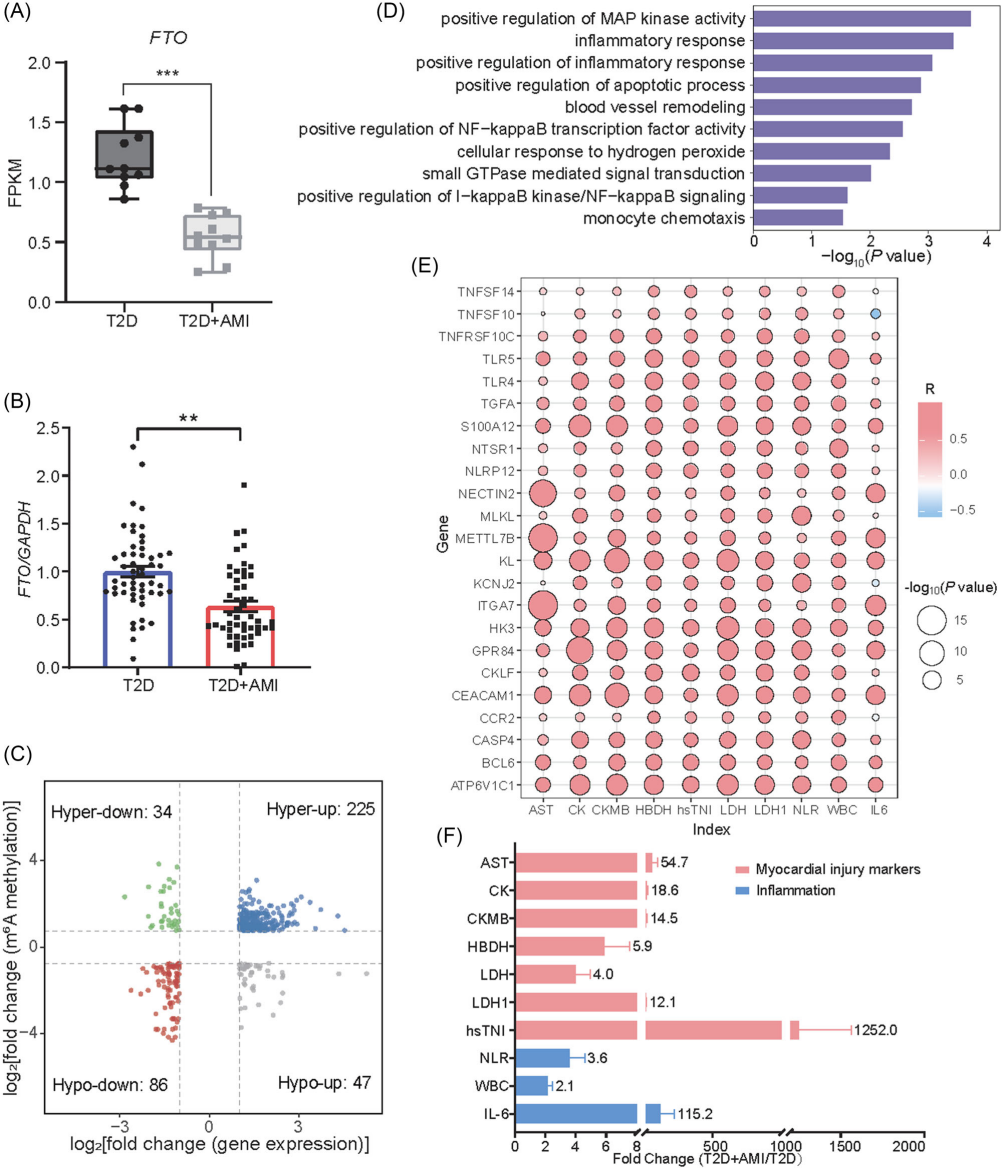
Characterization of differential m^6^A modifications between type 2 diabetes (T2D) and T2D+ acute myocardial infarction (AMI). (A) The expression of the *FTO* gene shows significant down‐regulation in patients with T2D+AMI, relative to patients with T2D (***p < 0.001). Bars represent mean ± SD. (B) *FTO* expression levels of T2D and T2D+AMI determined by real‐time quantitative polymerase chain reaction (RT‐qPCR) confirms the sequencing result (**p < 0.01). Bars represent mean ± SEM (standard error of the mean). (C) The four‐quadrant graph shows the distribution of genes with significant changes (|log_2_ (fold change)| ≥ 1, *p*  <  0.05) in expression and ≥ 1.68‐fold m^6^A alteration in patients with T2D+AMI compared to patients with T2D. (D) Gene ontology (GO) biological processes enriched among top differential genes with alterations in both gene expression and m^6^A between T2D+AMI and T2D. The *p*‐values are based on Fisher's exact test using the NIH/DAVID tool. (E) Correlation between candidate differential m^6^A genes and clinical indexes. R indicates the correlation coefficient. (F) Fold changes of clinical myocardial injury biomarkers and inflammation indicators in the T2D+AMI patients (n=10) compared to the T2D patients (n=10).

In summary, we developed SLIM‐m^6^A‐seq to implement transcriptome‐wide m^6^A profiling with limited RNA. Comprehensive analysis of SLIM‐m^6^A‐seq data in clinical samples from patients with T2D and T2D+AMI provided novel insights into the epitranscriptome of T2D+AMI regarding the contribution of m^6^A. Although with certain limitations (e.g. potential confounding due to immune cell composition), the overall m^6^A levels in T2D+AMI appeared to be elevated compared to T2D, accompanied by down‐regulation of *FTO*. Functional annotation analysis revealed relevant biological processes involved in the development of T2D+AMI. The current study demonstrated the technical robustness of SLIM‐m^6^A‐seq and importantly the feasibility of this new method in obtaining transcriptome‐wide m^6^A from clinically convenient samples, thus opening up opportunities for exploiting m^6^A as an effective clinical approach for disease diagnosis and prognosis.

## FUNDING INFORMATION

This work was supported by the National Natural Science Foundation of China (92253202, 91940304 and 22177087 to Xiaocheng Weng; 92153303, 22037004 and 91940000 to Xiang Zhou; 82172359, 81972009 and 81772276 to Song‐Mei Liu; and 22277094 to Fang Wang), Hubei Provincial Natural Science Fund for Creative Research Groups (2019CFA018), and Translational Medicine and Interdisciplinary Research Joint Fund of Zhongnan Hospital of Wuhan University (ZNJC201914).

## CONFLICT OF INTEREST

The authors declare that they have no conflict of interest.

## Supporting information

Supporting Information
