# Supplementary material for: N 6‐methyladenine profiling of low‐input multiplex clinical samples on transcriptome reveals RNA modifications implicated in type 2 diabetes and acute myocardial infarction
Source: Clin Transl Med. 2023 Jan 11;13(1):e1165. doi: 10.1002/ctm2.1165 (PMC9834504; doi:10.1002/ctm2.1165)
Supplement: Supplementary file 1 — Supporting Information [file CTM2-13-e1165-s001.docx]

Transcriptome-wide N^6^-methyladenine profiling in low-input multiplex clinical samples reveals RNA modifications implicated in type 2 diabetes and acute myocardial infarction

Shanshan Qin,^1,†^ Kun Yang,^2,†^ Shaoqing Han,^1,†^ Yushu Yuan,^1^ Jing Mo,^1^ Mengyao Xiao,^2^ Yin Yang,^2^ Yafen Wang,^1^ Xin Fang,^1^ Fang Wang,^3^ Wei Zhang,^4,*^, Song-Mei Liu,^2,*^ Xiaocheng Weng,^1,^* and Xiang Zhou^1,^*

^1^ College of Chemistry and Molecular Sciences, Key Laboratory of Biomedical Polymers of Ministry of Education, Wuhan University, Wuhan 430072, Hubei, China

^2^ Department of Clinical Laboratory, Center for Gene Diagnosis and Program of Clinical Laboratory, Zhongnan Hospital of Wuhan University, Wuhan 430071, Hubei, China

^3^ School of Pharmaceutical Sciences, Wuhan University, Wuhan 430071, Hubei, China

^4^ Department of Preventive Medicine, Northwestern University Feinberg School of Medicine, Chicago, Illinois 60611, USA

^†^These authors contributed equally.

*These authors contributed equally as senior authors.

**For correspondence:** Xiaocheng Weng, [xcweng@whu.edu.cn](mailto:xcweng@whu.edu.cn) College of Chemistry and Molecular Sciences, Key Laboratory of Biomedical Polymers of Ministry of Education, Wuhan University, Wuhan 430072, Hubei, China;

Song-Mei Liu, [smliu@whu.edu.cn](mailto:smliu@whu.edu.cn) Department of Clinical Laboratory, Center for Gene Diagnosis and Program of Clinical Laboratory, Zhongnan Hospital of Wuhan University, Wuhan 430071, Hubei, China;

Wei Zhang, [wei.zhang1@northwestern.edu](mailto:wei.zhang1@northwestern.edu) Department of Preventive Medicine, Northwestern University Feinberg School of Medicine, Chicago, Illinois 60611, USA;

Xiang Zhou, [xzhou@whu.edu.cn](mailto:xzhou@whu.edu.cn) College of Chemistry and Molecular Sciences, Key Laboratory of Biomedical Polymers of Ministry of Education, Wuhan University, Wuhan 430072, Hubei, China.

**Supporting Information**

**Supplementary Note 1**

**Data balance and independence of SLIM-m^6^A-seq**

To evaluate the balance of sequencing data across pooled samples, 3‘-linkers with different barcodes were tagged to 100 ng mRNA from HeLa cells separately, followed by pooling, m^6^A immunoprecipitation (IP) and library construction. The number of unique reads per library was analyzed, and we found comparable data percentages by different barcodes (Figure S1A). Furthermore, 320 ng of mRNA from HeLa cells was divided into 16 fractions using 16 unique 3‘-linkers, were profiled with SLIM-m^6^A-seq. The sequencing results suggested no significant biases in the read number between each barcode library, with 24.5% relative standard deviation (RSD) of m^6^A peak numbers (Figure S1B) and 21.83% RSD of gene read numbers (Figure S1C).

We then spiked four different artificial RNA oligonucleotides into four groups of mRNA samples and tagged each with an individual barcode to verify the independence of sequencing data (Figure S1D). The results showed that the spiked-in RNA oligonucleotides were detected only in the data split from their corresponding barcodes, thus confirming data independence and no data pollution of the proposed strategy (Figure S1E). The distribution of m^6^A on transcriptome explored by SLIM-m^6^A-seq matches the regular pattern (Figure S1F). Common RRACH motifs were also observed in each barcoded sample (Figure S1G).

**Supplementary Note 2**

**Validation of SLIM-m^6^A-seq in different amounts of RNA**

Two libraries were constructed to compare the data between conventional m^6^A-seq and SLIM-m^6^A-seq. One was constructed from a single sample of 800 ng of mRNA from HEK293T cells with m^6^A-seq. Another library was acquired by our strategy from a pooled sample with similar amount of total mRNA, including 11 barcoded samples varying from 200 ng to 10 ng (2 samples of 200 ng and 3 samples of 100 ng, 20 ng, 10 ng each). The results exhibited approximately 95% mapping rates (>70% uniquely mapped reads) on the transcriptome of HEK293T cells. m^6^A were enriched with approximately 4 folds in differently barcoded samples of different starting mRNA amounts (Figures S2B,C), with regular m^6^A distribution on transcriptome (Figure S2D). m^6^A profiles by SLIM-m^6^A-seq in samples containing no less than 100 ng mRNA suggested similar complexity compared to those by m^6^A-seq of 800 ng mRNA. In contrast, the SLIM-m^6^A-seq results of barcoded samples with 20 ng or 10 ng mRNA presented reduced complexity (Figure S3A). However, the reduced complexity can be attributed to the uneven distribution of read numbers resulted from simultaneous sequencing of different amounts of mRNA (Figure S3B). Additionally, SLIM-m^6^A-seq indicated a good correlation (R ≥ 0.76) between any two samples with similar complexity (distinct/total reads range from 0.82 to 0.86) regardless of the addition of barcode sequences, confirming the repeatability and reproducibility of our method (Figure S3C).

**Methods and Materials**

**Human blood sample collection**

A total of 135 patients were recruited from Zhongnan Hospital, including a cohort of 20 patients for SLIM-m^6^A-seq (T2D = 10, T2D+AMI = 10) and a cohort of 115 patients for HPLC-MS/MS and RT-qPCR (T2D = 64, T2D+AMI = 51). The AMI diagnosis was established according to the current universal definition of AMI at the time of the study.^1^ T2D was diagnosed following the Standards of Medical Care in Diabetes – 2017 of the American Diabetes Association.^2^ Patients were excluded if having hematological diseases, malignancy, severe renal or liver diseases, ongoing severe infection or chronic inflammatory diseases, and autoimmune diseases^3^. After an informed consent was obtained from each participant, fasting blood sample was collected for clinical laboratory tests (i.e., liver function, renal function, fasting glucose and lipids, cardiac biomarkers, inflammation) and RNA extraction on the morning following hospital admission. The details of clinical data are provided in Table S4 and Table S6. The distribution of age, sex, systolic blood pressure, and diastolic blood pressure in each cohort did not significantly differ between T2D and T2D+AMI patients (all *P* > 0.05). T2D+AMI patients exhibited increased cardiac biomarkers and inflammatory biomarkers compared to the respective upper reference limits (Figure S6).

**Cell culture**

HeLa and HEK293T cells obtained from American type culture collection (ATCC) were cultured using DMEM Medium (Gibco, USA) supplemented with 10% fetal bovine serum and 1% penicillin/streptomycin (Gibco, USA).

**SLIM-m^6^A-seq procedure**

*Pretreatment of 3’-linkers*

5 µL of 100 µM unmodified 3’-linkers were first phosphorylated by T4 polynucleotide kinase (PNK, NEB, USA; Lot: M0201L) at 37°C for 1 hour, and then the enzyme was deactivated at 65°C for 20 minutes. After purification by Oligo Clean & Concentrator (OCC, Zymo, USA; Lot: D4061) kit, phosphorylated 3’-linkers were eluted with 10 µL of RNase-free water. 3 µL of 5’-phosphorylated 3’-linkers were then adenylated using 5´ DNA adenylation kit (NEB, USA; Lot: E2610S). Finally, 30 µL of adenylated 3’-linkers were obtained after OCC kit purification. 5’-adenylated 3’-linkers were validated by 20% denaturing PAGE gel electrophoresis.

*mRNA isolation, fragmentation and PNK treatment*

Total RNA was extracted by TRIzol reagent (Invitrogen, USA; Lot: 15596018), and mRNA was then isolated using Oligo d(T)_25_ Magnetic Beads (NEB, USA; Lot: S1419S) and fragmented by Magnesium RNA Fragmentation Module (NEB, Country; Lot: E6150S). After purifying fragmented mRNAs with RNA Clean & Concentrator (RCC, Zymo, USA; Lot: R1016) kit, T4 PNK was subsequently used to remove 3' phosphoryl groups with the presence of RiboLock RNase Inhibitor (Thermofisher, USA; Lot: EO0381) in 10 uL reaction mix incubated at 37°C for 1 hour.

*3’-linker ligation*

Next, 2 µL of pre-adenylated 3’-linker, 1 µL of 10× T4 PNK Buffer, 6 µL of 50% PEG 8000, 1 µL of 0.1M DTT and 1 µL of T4 DNA ligase 2 truncated KQ (NEB, USA; Lot: M0373L) were added to the previous reaction mix and pipetted thoroughly. Incubation was performed at 25°C for 2 hours, followed by 16°C overnight. To remove excess 3’-linker, 1 µL of 5’-deadnylase (NEB, USA; Lot: M0331S) was added to the reaction mix and incubated at 30°C for 1 hour, followed by the addition of 1 µL of RecJf (NEB, USA; Lot: M0264L) and incubation for 1 hour at 37°C. The ligation products were purified using RCC kit and eluted with 50 µL of RNase-free water. 1 µL of eluted solution was retained as input sample and stored at -80°C.

*m^6^A immunoprecipitation*

m^6^A Immunoprecipitation (m^6^A IP) was implemented following the manufacturer’s protocol of the N^6^-Methyladenosine Enrichment Kit (NEB, USA; Lot: E1610S) to obtain IP samples. Reverse transcription was applied on both input and IP samples after denaturing at 75°C for 2 minutes with 1 µL of 2 µM RT primers. The reaction tube was transferred on ice immediately. Subsequently, 1 µL of RiboLock RNase Inhibitor, 4 µL of 5× First Strand Buffer, 1 µL of 0.1M DTT, 2 µL of 10mM dNTP mix (Thermofisher, USA; Lot: R0192), and 0.5 µL of SuperScript™ III Reverse Transcriptase (Invitrogen, USA; Lot: 18080044) were added to the reaction mix and incubated at 25°C for 3 minutes, 42°C for 10 minutes, 52°C for 40 minutes. To remove the excess primer, 1 µL of Exonuclease I (NEB, USA; Lot: M0293S) was added and incubated at 37°C for 30 minutes. RNA templates were excluded by adding 15 µL of 0.5M EDTA (pH 8.0) and 15 µL of 1M NaOH and incubating at 65°C for 15 minutes. cDNA strands were then purified using OCC kit and eluted with 7 µL of RNase-free water.

*5’-adaptor ligation and library construction*

Eluted cDNAs were denatured with 1 µL of DMSO and 0.3 µL of 80 µM 5’-adaptor at 75°C for 2 minutes. The reaction tube was transferred on ice immediately. Then 2 µL of 10× T4 RNA Ligation Buffer, 0.2 µL of 100mM ATP, 9 µL of 50% PEG 8000 and 1.5 µL of T4 RNA Ligase 1 (ssRNA Ligase), High Concentration (NEB, USA; Lot: M0437M) were added. After pipetting to mix thoroughly, the reaction mix was incubated at 25°C overnight. Next, cDNAs ligated with 5’-adaptor were purified using OCC kit and eluted with 12 µL of RNase-free water. qPCR was performed to determine the number of library PCR cycles. Finally, library PCR was performed using Ultra™ II Q5® Master Mix (NEB, USA; Lot: M0544S) and Multiplex Oligos for Illumina® (NEB, Country; Lot: E7335S) following the manufacturer’s protocol. All primer sequences are shown in Table S1.

**Sequencing data processing**

The genome references (human hg38 and mouse mm10) and a list of transcripts (human transcripts v31 and mouse transcripts v24) were downloaded from Gencode.^4^ Raw FASTQ reads were trimmed to remove adaptors and aligned to the reference genomes using *cutadapt*^5^ and STAR 2.7.5a,^6^ respectively. Complexity was evaluated by *preseq* from 1 million sampled reads or the maximum reads number.^7^ Aligned reads were used for peak calling and enriched regions comparing by *exomePeak.* HOMER was used to detect sequence motifs.

**Differential gene and pathway analysis**

Differentially expressed genes were identified using DESeq2^8^ with the cutoffs of adjusted *P* < 0.05 estimated using the Benjamini-Hochberg procedure^9^ and log_2_(Fold Change) > 1. We evaluated the relative importance of differential features by applying the elastic net regularization on logistic linear regression models using the *glmnet* library in R Statistical Environment,^10^ where the Lasso penalty was set as α = 0.5, and carried out the Leave-One-Out Cross Validation (LOOCV) to select robust differential genes that were selected in at least 15 out of 20 times of validation. The NIH/DAVID tool was used for Gene Ontology (GO) enrichment analysis.^11^

**Determining m^6^A/A with HPLC-MS/MS analysis**

Poly(A)+ RNA was enriched twice and then processed as previously described.^12^ Analysis of nucleosides was performed by the Shimadzu LCMS-8050 system (Shimadzu, Kyoto, Japan) under the multiple reaction monitoring mode using retention time and nucleoside to base ion mass transitions of 282.1 to 150.1 (m^6^A), and 268 to 136 (A). The coefficient values (R^2^) were higher than 0.99.

**RT-qPCR**

RT-qPCR was used to determine the expression of mRNA in validation samples on a CFX‐96 Real‐Time System (Bio‐Rad, USA). Primers are listed in Table S7.

**Statistical analysis**

All statistical analyses were performed using the SPSS 21.0 (IBM Inc., Chicago, IL, USA) or the R Statistical Environment.^13^ The χ^2^ test was used to evaluate whether sex and age distributions were balanced in all participants. The Shapiro–Wilk test was used to exam data normality. The two-tailed, unpaired Student’s t test was used to assess the statistical significance of differences between two sets of normally distributed data. The nonparametric Mann-Whitney U test was performed for skewed data. The results of clinical variables are expressed as mean ± SD (standard deviation) or median (IQR). Gene expression levels in RT-qPCR are presented as mean ± S.E.M. (satndard error of the mean, n = 3). In HPLC-MS/MS experiments, each experiment was performed in triplicates and the number of asterisks is used to designate the following levels of statistical significance: ****P* < 0.001, ***P* < 0.01, **P* < 0.05 compared to the control group.

**References**

1. Thygesen K, Alpert JS, Jaffe AS, et al. Third universal definition of myocardial infarction. *Eur Heart J.* 2012;33(20):2551-2567.

2. Standards of Medical Care in Diabetes-2017: Summary of Revisions. *Diabetes Care.* 2017;40 (Suppl 1):S4-S5.

3. Steg PG, Bhatt DL, Simon T, et al. Ticagrelor in Patients with Stable Coronary Disease and Diabetes. *N Engl J Med*. 2019;381(14):1309-1320.

4. Harrow J, Frankish A, Gonzalez J M, et al. GENCODE: the reference human genome annotation for The ENCODE Project. *Genome Res.* 2012; 22(9):1760–1774.

5. Kechin A, Boyarskikh U, Kel A, Filipenko M. CutPrimers: a new tool for accurate cutting of primers from reads of targeted next generation sequencing. *J Comput Biol.* 2017;24(11):1138-1143.

6. Dobin A, Davis CA, Schlesinger F, et al. STAR: ultrafast universal RNA-seq aligner. *Bioinformatics*. 2013;29(1):15-21.

7. Daley T, Smith AD. Predicting the molecular complexity of sequencing libraries. *Nat Methods.* 2013;10(4):325-327.

8. Love MI, Huber W, Anders S. Moderated estimation of fold change and dispersion for RNA-seq data with DESeq2. *Genome Biol.* 2014;15(12):550.

9. Benjamini Y, Hochberg J. Controlling the False Discovery Rate: A Practical and Powerful Approach to Multiple Testing. *Journal of the Royal Statistical Society: Series B* 1995;57(No. 1):289–300.

10. Friedman J, Hastie T, Tibshirani R. Regularization Paths for Generalized Linear Models via Coordinate Descent. *J Stat Softw.* 2010;33(1):1-22.

11. Jiao X, Sherman BT, Huang da W, et al. DAVID-WS: a stateful web service to facilitate gene/protein list analysis. *Bioinformatics*. 2012;28(13):1805-1806.

12. Dominissini D, Moshitch-Moshkovitz S, Schwartz S, et al. Topology of the human and mouse m^6^A RNA methylomes revealed by m^6^A-seq. *Nature.* 2012;485 (7397):201-206.

13. IBM Corp. Released 2013. IBM SPSS Statistics for Windows, Version 22.0. Armonk, NY: IBM Corp.

**Supplementary Figures**


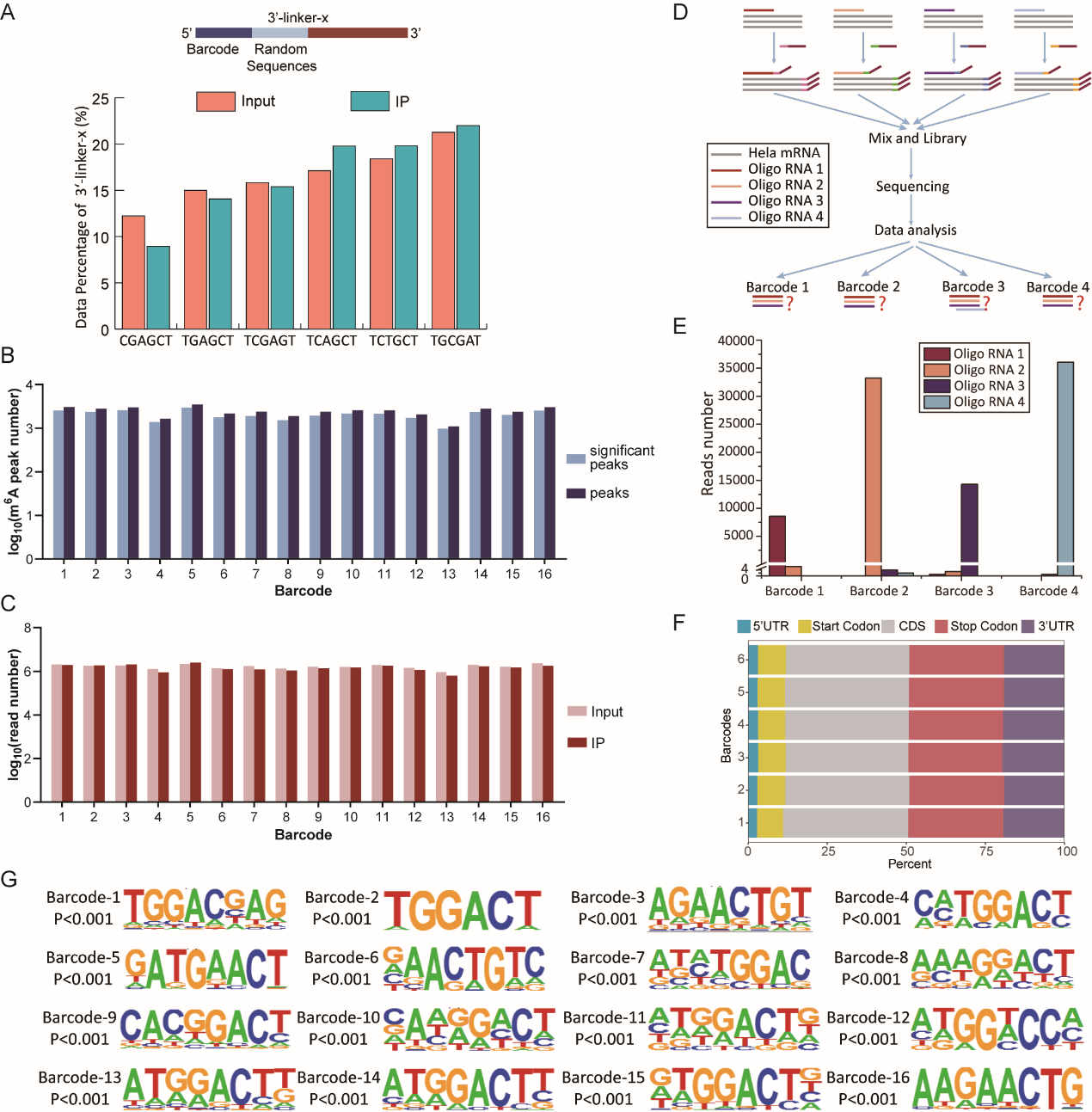


**Figure S1. Performance of SLIM-m^6^A-seq on the aspect of sequencing data balance and independence. A** Data percentage of different samples labeled with 3’-linker-x. 6-nt sequences on x axis are the expected barcode sequence reads according to which sequencing data were split. **B** The numbers of detected m^6^A peaks in 16 ~~IP~~ fractions from 320 ng HeLa cell mRNA separated by different barcodes are shown by log of 10. Total m^6^A peaks are in dark blue. Significant m^6^A peaks with fold enrichment > 2 are in light blue. **C** The total read numbers of the input (light red) and IP (dark red) samples in the same 16 fractions of HeLa cells are shown by log of 10. IP: immunoprecipitation. **D** Scheme of data independence test. **E** Results of data independence test. A break is made from 5 to 10 at 4% of y axis length. **F** m^6^A distributions of samples barcoded with 3‘-linker-1~6 were analysed and shown in percentages of different Regions on transcriptome. Barcodes 1~6 on the y axis correspond to 3‘-linker-1~6. UTR: untranslated region; CDS: coding sequence. **G** Top sequence motifs found in each barcoded samples within enriched m^6^A peaks identified by SLIM-m^6^A-seq. Barcode-1~16 correspond to the barcode sequences of 3‘-linker-1~16 shown in Table S1.


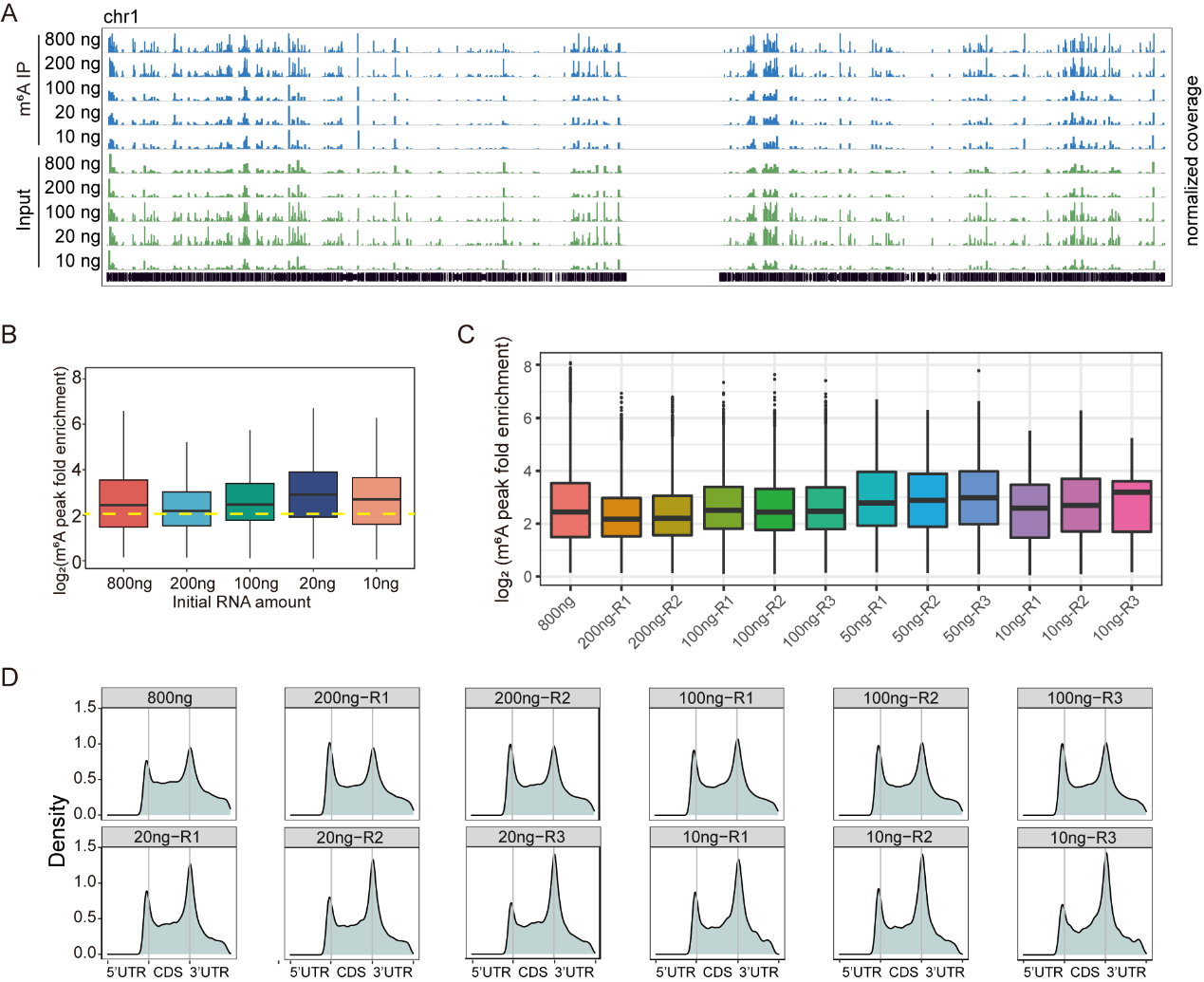


**Figure S2. Validation of SLIM-m^6^A-seq in serial input materials. A** Chromosome 1 is shown as an example for consistent m^6^A profiles across different samples. **B** The box plot shows comparable m^6^A fold enrichment values for a series of SLIM-m^6^A-seq samples (10 ng to 200 ng) and the m^6^A-seq reference (800 ng). **C** Box plot showing detailed m^6^A fold enrichments in each sample. Center line, median; box limits, upper and lower quartiles; whiskers, 1.5× interquartile range; points, outliers. **D** The density plots show consistent distribution patterns of m^6^A peaks between a series of SLIM-m^6^A-seq samples (10 ng to 200 ng) and the m^6^A-seq reference (800 ng). UTR: untranslated region; CDS: coding sequences.


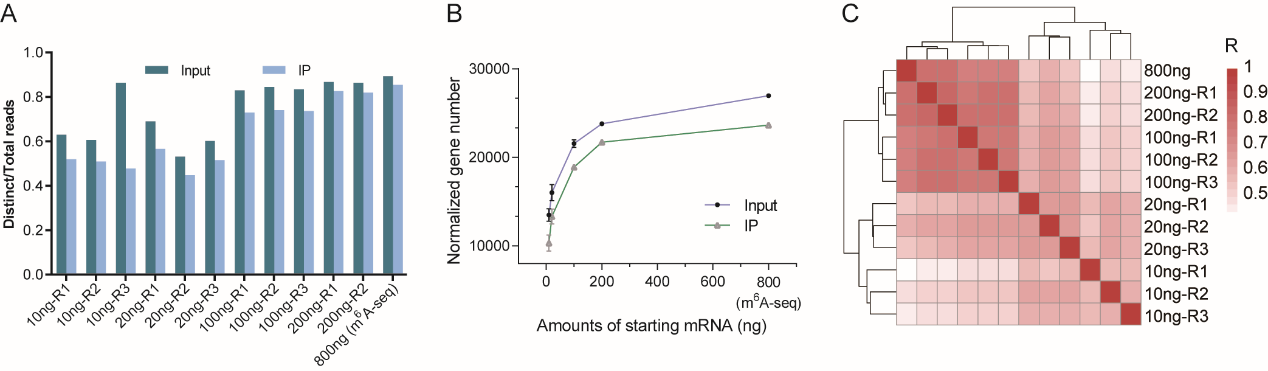


**Figure S3. Complexities and correlations of library constructed by SLIM-m^6^A-seq and m^6^A-seq. A** Sequencing complexity is shown by the ratio of distinct reads to total reads in a series of initial RNA amounts. **B** Normalized gene numbers of the m^6^A-seq sample and SLIM-m^6^A-seq samples with different initial RNA amounts. **C** The hierarchical clustering shows correlation between m^6^A profiles from a series of input amounts using SLIM-m^6^A-seq (10 ng to 200 ng) and the m^6^A-seq reference (800 ng).


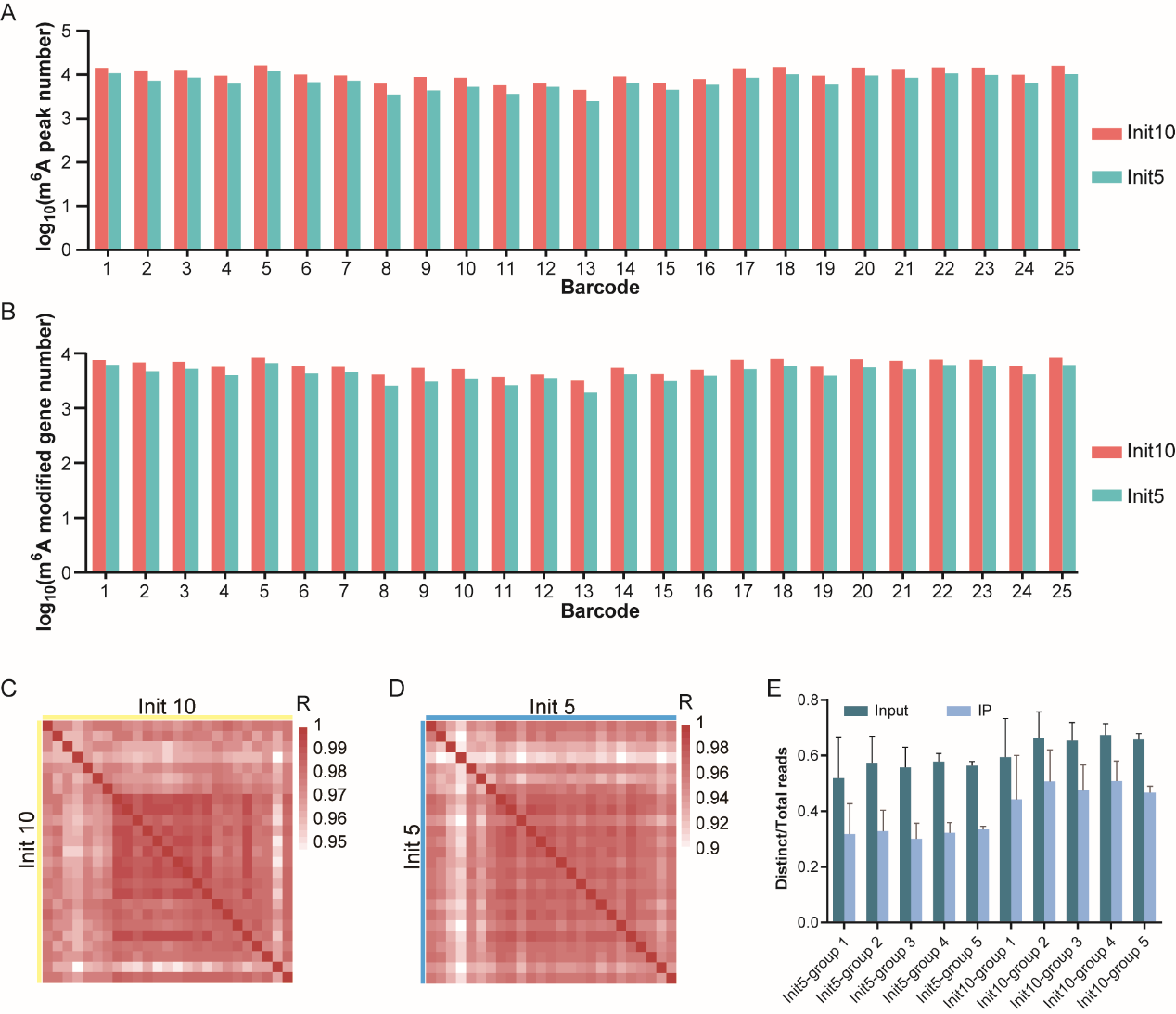


**Figure S4. SLIM-m^6^A-seq in low-input samples. A** m^6^A peaks found in sequencing data of individually barcoded samples split from Init10 (orange) and Init5 (cyan). **B** Detected gene numbers containing m^6^A modifications in individually barcoded samples split from Init10 (orange) and Init5 (cyan). **C** Correlation of m^6^A profiles in 25 differently barcoded samples, each contains 10 ng HEK293T cell mRNA. These samples were pooled and sequenced as Init10. **D** Correlation of m^6^A profiles in 25 differently barcoded samples, each contains 5 ng HEK293T cell mRNA. These samples were pooled and sequenced as Init5. Hierarchical clustering applied to the matrix of sample correlations based on detected m^6^A enriched peaks is shown in **C** and **D**. **E** Sequencing complexity is shown by the ratio of distinct reads to total reads. The 25 barcoded samples of Init5 (5ng RNA) or Init10 (10ng RNA), respectively, are combined into five groups (5 samples/group) for the calculation of complexity.


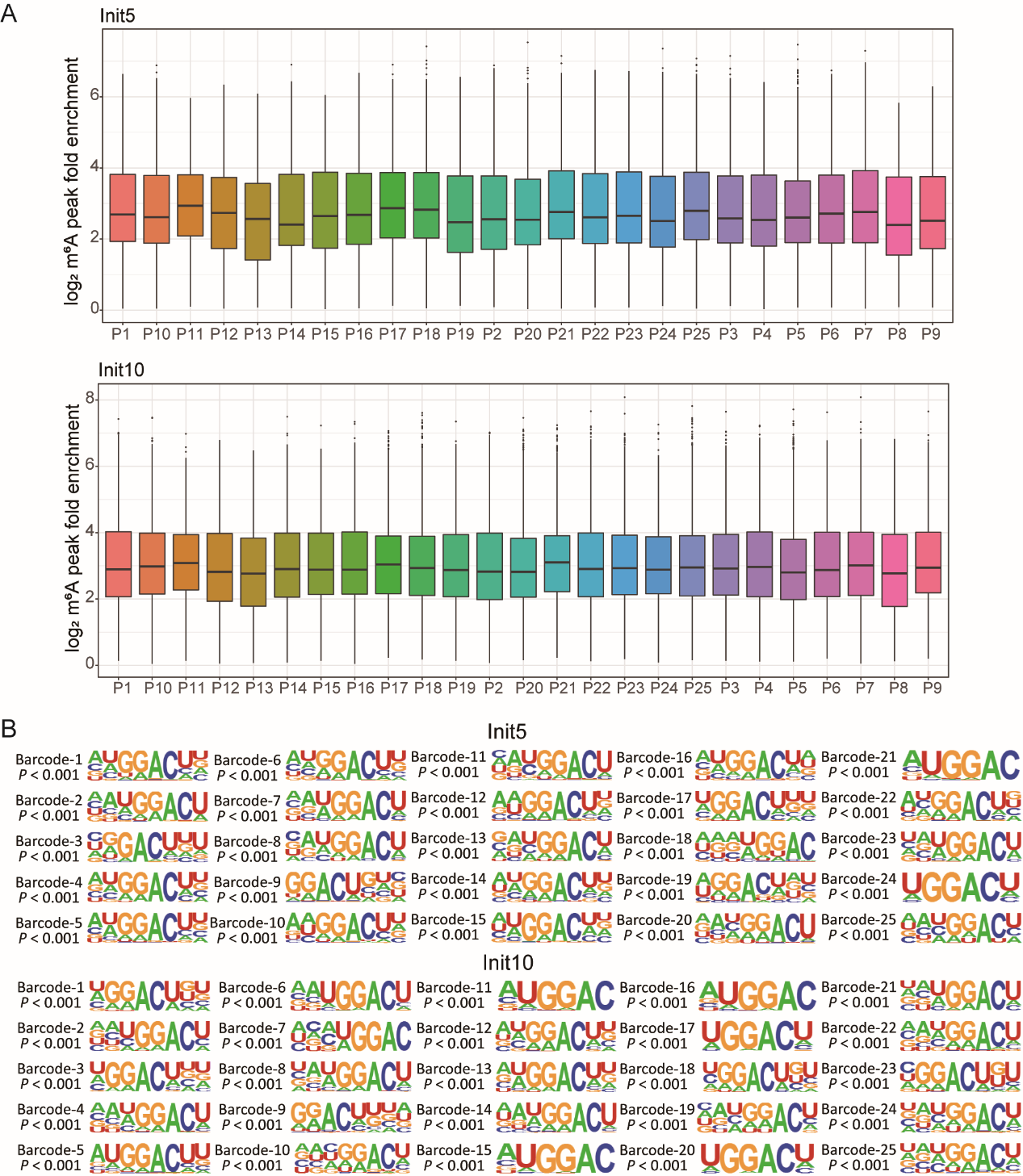


**Figure S5.** **m^6^A fold enrichments and motifs** **in individually barcoded samples split from Init10 and Init5. A** m^6^A fold enrichments in individually barcoded samples contained in Init 5 (upper) and Init10 (lower), with the median value of m^6^A fold enrichment range from 5.25 to 7.54 in Init5 and 6.79 to 8.58 in Init 10. Labels on the x axis: P1~P25 correspond to pooled samples with different barcode labels (see Table S1). Center line, median; box limits, upper and lower quartiles; whiskers, 1.5× interquartile range; points, outliers. **B** Top sequence motifs and corresponding *P* values of m^6^A enriched region in individually barcoded samples split from Init5 and Init10 identified by SLIM-m^6^A-seq.


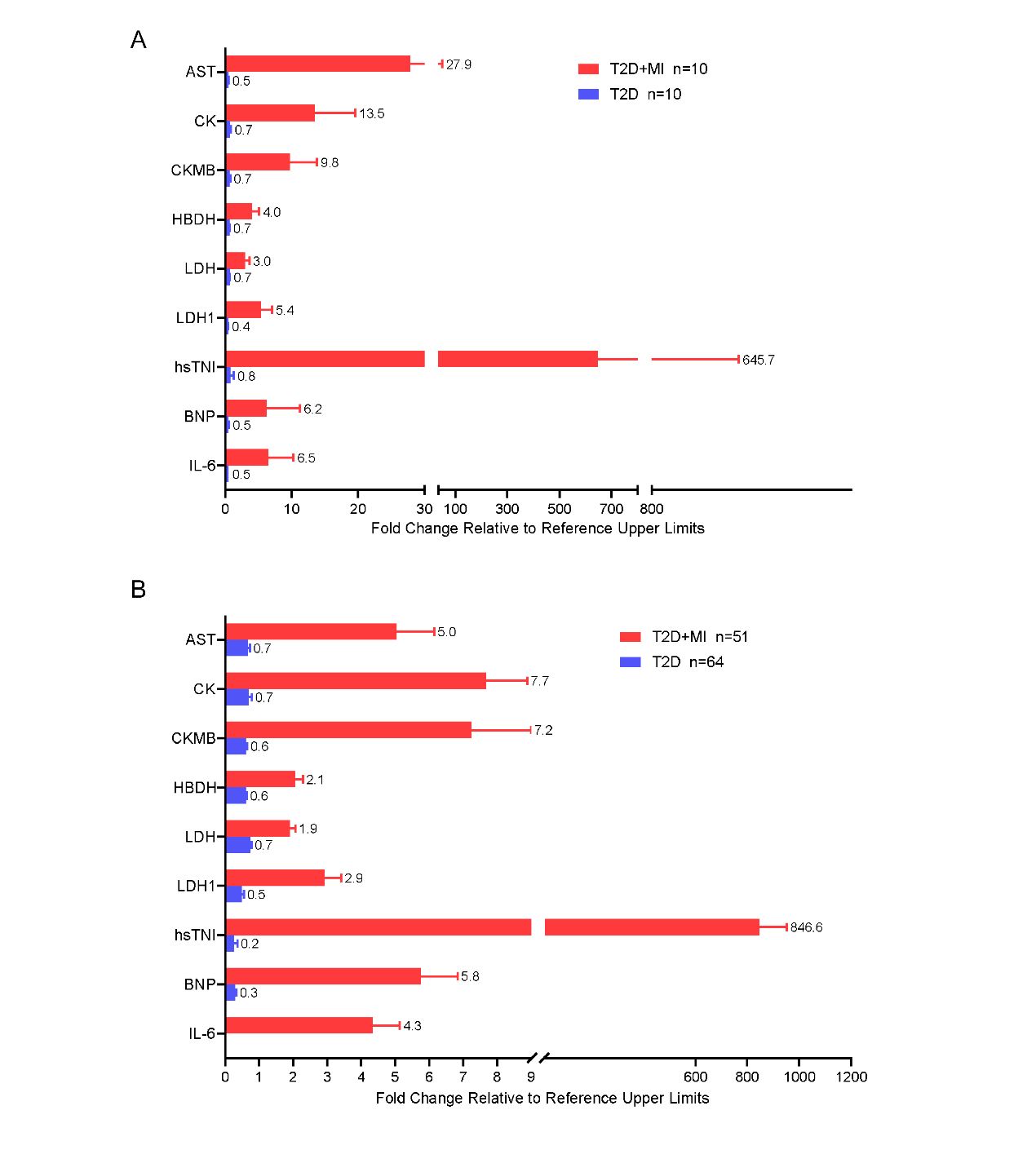


**Figure S6.** **Elevation of clinical myocardial injury biomarkers and inflammation indicators in T2D+AMI patients**. **A** Fold changes of clinical parameters in T2D and T2D+AMI patients for SLIM-m^6^A-seq compared to their upper reference limits. **B** Fold changes of clinical parameters in T2D and T2D+AMI patients for HPLC-MS/MS and RT-qPCR compared to their upper reference limits.

**Supplementary Tables**

**Table S1. Oligonucleotides used in library construction.**

| Name | Sequence (from 5’ to 3’) |
| --- | --- |
| 3’-linker-1^a)^ | **ACTCGA***NNNNNN*AGATCGGAAGAGCGTCGTG-SpC3 |
| 3’-linker-2 | **AGCTGA***NNNNNN*AGATCGGAAGAGCGTCGTG-SpC3 |
| 3’-linker-3 | **AGCAGA***NNNNNN*AGATCGGAAGAGCGTCGTG-SpC3 |
| 3’-linker-4 | **AGCTCG***NNNNNN*AGATCGGAAGAGCGTCGTG-SpC3 |
| 3’-linker-5 | **ATCGCA***NNNNNN*AGATCGGAAGAGCGTCGTG-SpC3 |
| 3’-linker-6 | **AGCTCA***NNNNNN*AGATCGGAAGAGCGTCGTG-SpC3 |
| 3’-linker-7 | **TTCGGA***NNNNNN*AGATCGGAAGAGCGTCGTG-SpC3 |
| 3’-linker-8 | **CATCGA***NNNNNN*AGATCGGAAGAGCGTCGTG-SpC3 |
| 3’-linker-9 | **CTAGCA***NNNNNN*AGATCGGAAGAGCGTCGTG-SpC3 |
| 3’-linker-10 | **ACGGTA***NNNNNN*AGATCGGAAGAGCGTCGTG-SpC3 |
| 3’-linker-11 | **CCATTG***NNNNNN*AGATCGGAAGAGCGTCGTG-SpC3 |
| 3’-linker-12 | **GATTCG***NNNNNN*AGATCGGAAGAGCGTCGTG-SpC3 |
| 3’-linker-13 | **CGTTAG***NNNNNN*AGATCGGAAGAGCGTCGTG-SpC3 |
| 3’-linker-14 | **GACTGT***NNNNNN*AGATCGGAAGAGCGTCGTG-SpC3 |
| 3’-linker-15 | **ACAGCA***NNNNNN*AGATCGGAAGAGCGTCGTG-SpC3 |
| 3’-linker-16 | **AGTCGT***NNNNNN*AGATCGGAAGAGCGTCGTG-SpC3 |
| RT primer | ACACGACGCTCTTCCGATCT |
| 5’-adaptor ^b)^ | Phos-*NNNNNNNNNN*AGATCGGAAGAGCACACGTCTG-SpC3 |
| Forward primer for qPCR | ACACGACGCTCTTCCGATCT |
| Reverse primer for qPCR | CAGACGTGTGCTCTTCCGAT |
| Library PCR forward primer (for NGS) | AATGATACGGCGACCACCGAGATCTACACTCTTTCCCTACACGACGCTCTTCCGATC*T |
| Library PCR reverse primer (for NGS) ^c)^ | CAAGCAGAAGACGGCATACGAGAT**NNNNNN**GTGACTGGAGTTCAGACGTGTGCTCTTCCGATC-s-T |
| Oligo RNA 1 | AUACUGCCACAUGCUGCACAGUGC |
| Oligo RNA 2 | GGACUGAGAACUGGACUGUCUGGGGUGCCAAGGUA |
| Oligo RNA 3 | GGACUGAACUGGACUGUCUGGGGUGCCAAGGUA |
| Oligo RNA 4 | GUACGUCAUCGAGAUCAGCUU |

1. 6-nt sequences displayed in bold are barcode sequences. b) sequences comprise of Italic “*N*” represent random sequences of different length. c) 6-nt sequence comprise of bold “**N**” with underline represents the index sequence contained in library PCR primer for next generation sequencing (NGS).

**Table S2. Expected barcode reads corresponding to individual samples.**

| No.^a)^ | Species | Sample Type | Included Individual Sample ^b)^ | Individual Sample mRNA Amount | 3’-linker-x Used | Expected Barcode Reads ^c)^ |
| --- | --- | --- | --- | --- | --- | --- |
| 1 | Homo sapiens | HEK293T Cell | 200 ng-rep 1 | 200 ng | 3’-linker-9 | TGCTAG |
|  | Homo sapiens | HEK293T Cell | 200 ng-rep 2 | 200 ng | 3’-linker-10 | TACCGT |
|  | Homo sapiens | HEK293T Cell | 100 ng-rep 1 | 100 ng | 3’-linker-12 | CGAATC |
|  | Homo sapiens | HEK293T Cell | 100 ng-rep 2 | 100 ng | 3’-linker-14 | ACAGTC |
|  | Homo sapiens | HEK293T Cell | 100 ng-rep 3 | 100 ng | 3’-linker-16 | ACGACT |
|  | Homo sapiens | HEK293T Cell | 20 ng-rep 1 | 20 ng | 3’-linker-5 | TGCGAT |
|  | Homo sapiens | HEK293T Cell | 20 ng-rep 2 | 20 ng | 3’-linker-6 | TGAGCT |
|  | Homo sapiens | HEK293T Cell | 20 ng-rep 3 | 20 ng | 3’-linker-7 | TCCGAA |
|  | Homo sapiens | HEK293T Cell | 10 ng-rep 1 | 10 ng | 3’-linker-2 | TCAGCT |
|  | Homo sapiens | HEK293T Cell | 10 ng-rep 2 | 10 ng | 3’-linker-3 | TCTGCT |
|  | Homo sapiens | HEK293T Cell | 10 ng-rep 3 | 10 ng | 3’-linker-4 | CGAGCT |
| 2 | Homo sapiens | HEK293T Cell | Init10_1 | 10 ng | 3’-linker-1 | ACTCGA |
|  | Homo sapiens | HEK293T Cell | Init10_2 | 10 ng | 3’-linker-2 | AGCTGA |
|  | Homo sapiens | HEK293T Cell | Init10_3 | 10 ng | 3’-linker-3 | AGCAGA |
|  | Homo sapiens | HEK293T Cell | Init10_4 | 10 ng | 3’-linker-4 | AGCTCG |
|  | Homo sapiens | HEK293T Cell | Init10_5 | 10 ng | 3’-linker-5 | ATCGCA |
|  | Homo sapiens | HEK293T Cell | Init10_6 | 10 ng | 3’-linker-6 | AGCTCA |
|  | Homo sapiens | HEK293T Cell | Init10_7 | 10 ng | 3’-linker-7 | TTCGGA |
|  | Homo sapiens | HEK293T Cell | Init10_8 | 10 ng | 3’-linker-8 | CATCGA |
|  | Homo sapiens | HEK293T Cell | Init10_9 | 10 ng | 3’-linker-9 | CTAGCA |
|  | Homo sapiens | HEK293T Cell | Init10_10 | 10 ng | 3’-linker-10 | ACGGTA |
|  | Homo sapiens | HEK293T Cell | Init10_11 | 10 ng | 3’-linker-11 | CCATTG |
|  | Homo sapiens | HEK293T Cell | Init10_12 | 10 ng | 3’-linker-12 | GATTCG |
|  | Homo sapiens | HEK293T Cell | Init10_13 | 10 ng | 3’-linker-13 | CGTTAG |
|  | Homo sapiens | HEK293T Cell | Init10_14 | 10 ng | 3’-linker-14 | GACTGT |
|  | Homo sapiens | HEK293T Cell | Init10_15 | 10 ng | 3’-linker-15 | ACAGCA |
|  | Homo sapiens | HEK293T Cell | Init10_16 | 10 ng | 3’-linker-16 | AGTCGT |
|  | Homo sapiens | HEK293T Cell | Init10_17 | 10 ng | 3’-linker-17 | GACAGT |
|  | Homo sapiens | HEK293T Cell | Init10_18 | 10 ng | 3’-linker-18 | CTGCAT |
|  | Homo sapiens | HEK293T Cell | Init10_19 | 10 ng | 3’-linker-19 | AGAGCT |
|  | Homo sapiens | HEK293T Cell | Init10_20 | 10 ng | 3’-linker-20 | GCAGTT |
|  | Homo sapiens | HEK293T Cell | Init10_21 | 10 ng | 3’-linker-21 | GAACCT |
|  | Homo sapiens | HEK293T Cell | Init10_22 | 10 ng | 3’-linker-22 | GTGATC |
|  | Homo sapiens | HEK293T Cell | Init10_23 | 10 ng | 3’-linker-24 | GATTGC |
|  | Homo sapiens | HEK293T Cell | Init10_24 | 10 ng | 3’-linker-25 | AGACTC |
|  | Homo sapiens | HEK293T Cell | Init10_25 | 10 ng | 3’-linker-26 | CTATGG |
| 3 | Homo sapiens | HEK293T Cell | Init5_1 | 5 ng | 3’-linker-1 | ACTCGA |
|  | Homo sapiens | HEK293T Cell | Init5_2 | 5 ng | 3’-linker-2 | AGCTGA |
|  | Homo sapiens | HEK293T Cell | Init5_3 | 5 ng | 3’-linker-3 | AGCAGA |
|  | Homo sapiens | HEK293T Cell | Init5_4 | 5 ng | 3’-linker-4 | AGCTCG |
|  | Homo sapiens | HEK293T Cell | Init5_5 | 5 ng | 3’-linker-5 | ATCGCA |
|  | Homo sapiens | HEK293T Cell | Init5_6 | 5 ng | 3’-linker-6 | AGCTCA |
|  | Homo sapiens | HEK293T Cell | Init5_7 | 5 ng | 3’-linker-7 | TTCGGA |
|  | Homo sapiens | HEK293T Cell | Init5_8 | 5 ng | 3’-linker-8 | CATCGA |
|  | Homo sapiens | HEK293T Cell | Init5_9 | 5 ng | 3’-linker-9 | CTAGCA |
|  | Homo sapiens | HEK293T Cell | Init10_10 | 5 ng | 3’-linker-10 | ACGGTA |
|  | Homo sapiens | HEK293T Cell | Init10_11 | 5 ng | 3’-linker-11 | CCATTG |
|  | Homo sapiens | HEK293T Cell | Init10_12 | 5 ng | 3’-linker-12 | GATTCG |
|  | Homo sapiens | HEK293T Cell | Init10_13 | 5 ng | 3’-linker-13 | CGTTAG |
|  | Homo sapiens | HEK293T Cell | Init10_14 | 5 ng | 3’-linker-14 | GACTGT |
|  | Homo sapiens | HEK293T Cell | Init10_15 | 5 ng | 3’-linker-15 | ACAGCA |
|  | Homo sapiens | HEK293T Cell | Init10_16 | 5 ng | 3’-linker-16 | AGTCGT |
|  | Homo sapiens | HEK293T Cell | Init10_17 | 5 ng | 3’-linker-17 | GACAGT |
|  | Homo sapiens | HEK293T Cell | Init10_18 | 5 ng | 3’-linker-18 | CTGCAT |
|  | Homo sapiens | HEK293T Cell | Init10_19 | 5 ng | 3’-linker-19 | AGAGCT |
|  | Homo sapiens | HEK293T Cell | Init10_20 | 5 ng | 3’-linker-20 | GCAGTT |
|  | Homo sapiens | HEK293T Cell | Init10_21 | 5 ng | 3’-linker-21 | GAACCT |
|  | Homo sapiens | HEK293T Cell | Init10_22 | 5 ng | 3’-linker-22 | GTGATC |
|  | Homo sapiens | HEK293T Cell | Init10_23 | 5 ng | 3’-linker-24 | GATTGC |
|  | Homo sapiens | HEK293T Cell | Init10_24 | 5 ng | 3’-linker-25 | AGACTC |
|  | Homo sapiens | HEK293T Cell | Init10_25 | 5 ng | 3’-linker-26 | CTATGG |
| 4 | Homo sapiens | Peripheral Blood | T2D-1 | 160 ng | 3’-linker-1 | TCGAGT |
|  | Homo sapiens | Peripheral Blood | T2D-2 | 160 ng | 3’-linker-2 | TCAGCT |
|  | Homo sapiens | Peripheral Blood | T2D-3 | 160 ng | 3’-linker-3 | TCTGCT |
|  | Homo sapiens | Peripheral Blood | T2D-4 | 160 ng | 3’-linker-4 | CGAGCT |
|  | Homo sapiens | Peripheral Blood | T2D-5 | 160 ng | 3’-linker-5 | TGCGAT |
|  | Homo sapiens | Peripheral Blood | T2D-6 | 160 ng | 3’-linker-6 | TGAGCT |
|  | Homo sapiens | Peripheral Blood | T2D-7 | 160 ng | 3’-linker-7 | TCCGAA |
|  | Homo sapiens | Peripheral Blood | T2D-8 | 160 ng | 3’-linker-8 | TCGATG |
|  | Homo sapiens | Peripheral Blood | T2D-9 | 160 ng | 3’-linker-9 | TGCTAG |
|  | Homo sapiens | Peripheral Blood | T2D-10 | 160 ng | 3’-linker-10 | TACCGT |
| 5 | Homo sapiens | Peripheral Blood | T2D+AMI-1 | 160 ng | 3’-linker-1 | TCGAGT |
|  | Homo sapiens | Peripheral Blood | T2D+AMI-2 | 160 ng | 3’-linker-2 | TCAGCT |
|  | Homo sapiens | Peripheral Blood | T2D+AMI-3 | 160 ng | 3’-linker-3 | TCTGCT |
|  | Homo sapiens | Peripheral Blood | T2D+AMI-4 | 160 ng | 3’-linker-4 | CGAGCT |
|  | Homo sapiens | Peripheral Blood | T2D+AMI-5 | 160 ng | 3’-linker-5 | TGCGAT |
|  | Homo sapiens | Peripheral Blood | T2D+AMI-6 | 160 ng | 3’-linker-6 | TGAGCT |
|  | Homo sapiens | Peripheral Blood | T2D+AMI-7 | 160 ng | 3’-linker-7 | TCCGAA |
|  | Homo sapiens | Peripheral Blood | T2D+AMI-8 | 160 ng | 3’-linker-8 | TCGATG |
|  | Homo sapiens | Peripheral Blood | T2D+AMI-9 | 160 ng | 3’-linker-9 | TGCTAG |
|  | Homo sapiens | Peripheral Blood | T2D+AMI-10 | 160 ng | 3’-linker-10 | TACCGT |

a) No.1-5 suggest 5 pooled samples containing different individual samples. b) Clinical baseline characteristics of peripheral blood samples of T2D/T2D+AMI are shown in Table S4. c) Expected barcode reads in sequencing data. Individual samples were labelled with 3’-linkers before pooling and performing SLIM-m^6^A-seq, corresponding expected barcode reads of each 3’-linker are shown.

**Table S3. Sequencing analysis summary of individually barcoded samples split from Init10 and Init5.**

| individual sample | treatment | Uniquely mapped | Uniquely mapped | Total input reads | Overall mapping rate | Average read length |
| --- | --- | --- | --- | --- | --- | --- |
| Init10_10 | none | 1083416 | 68.08% | 1591415 | 93.50% | 120 |
| Init10_11 |  | 692841 | 65.51% | 1057638 | 91.68% | 114 |
| Init10_12 |  | 893558 | 65.41% | 1365997 | 91.24% | 114 |
| Init10_13 |  | 649871 | 66.39% | 978934 | 92.15% | 118 |
| Init10_14 |  | 1198056 | 67.99% | 1762164 | 93.09% | 117 |
| Init10_15 |  | 757242 | 67.44% | 1122800 | 93.28% | 118 |
| Init10_16 |  | 1105613 | 67.53% | 1637215 | 92.79% | 118 |
| Init10_17 |  | 1935426 | 70.11% | 2760716 | 94.60% | 120 |
| Init10_18 |  | 2262520 | 69.83% | 3240008 | 94.46% | 119 |
| Init10_19 |  | 1392110 | 69.86% | 1992835 | 94.59% | 120 |
| Init10_1 |  | 2301949 | 69.11% | 3330718 | 94.21% | 118 |
| Init10_20 |  | 2313009 | 69.91% | 3308493 | 94.53% | 120 |
| Init10_21 |  | 1750818 | 69.94% | 2503329 | 94.60% | 120 |
| Init10_22 |  | 2438597 | 70.03% | 3482119 | 94.51% | 120 |
| Init10_23 |  | 2193581 | 70.04% | 3131949 | 94.38% | 121 |
| Init10_24 |  | 1316378 | 69.33% | 1898730 | 94.68% | 118 |
| Init10_25 |  | 2471780 | 70.13% | 3524678 | 94.57% | 121 |
| Init10_2 |  | 2015105 | 69.88% | 2883600 | 94.61% | 120 |
| Init10_3 |  | 1834719 | 69.68% | 2633104 | 94.31% | 120 |
| Init10_4 |  | 1378469 | 69.12% | 1994235 | 94.47% | 119 |
| Init10_5 |  | 2840782 | 69.15% | 4108224 | 94.23% | 119 |
| Init10_6 |  | 1389774 | 69.00% | 2014136 | 94.29% | 118 |
| Init10_7 |  | 1328659 | 67.18% | 1977842 | 93.02% | 117 |
| Init10_8 |  | 950791 | 66.28% | 1434415 | 91.74% | 115 |
| Init10_9 |  | 1178942 | 67.90% | 1736351 | 92.93% | 119 |
| Init10_10 | m^6^A IP | 758979 | 77.72% | 976540 | 92.52% | 130 |
| Init10_11 |  | 515549 | 78.13% | 659862 | 92.52% | 132 |
| Init10_12 |  | 462937 | 77.70% | 595784 | 91.98% | 131 |
| Init10_13 |  | 301288 | 77.92% | 386642 | 92.22% | 131 |
| Init10_14 |  | 721108 | 78.13% | 922919 | 92.42% | 129 |
| Init10_15 |  | 573849 | 77.56% | 739907 | 92.69% | 128 |
| Init10_16 |  | 737889 | 77.46% | 952631 | 92.37% | 131 |
| Init10_17 |  | 1918381 | 77.96% | 2460868 | 93.38% | 130 |
| Init10_18 |  | 2100951 | 77.96% | 2694902 | 93.48% | 131 |
| Init10_19 |  | 951184 | 77.59% | 1225861 | 93.47% | 130 |
| Init10_1 |  | 1718950 | 78.32% | 2194680 | 93.53% | 129 |
| Init10_20 |  | 1864399 | 77.63% | 2401792 | 93.34% | 129 |
| Init10_21 |  | 1924409 | 77.97% | 2468086 | 93.58% | 130 |
| Init10_22 |  | 1819135 | 78.24% | 2325140 | 93.41% | 131 |
| Init10_23 |  | 1812690 | 78.15% | 2319608 | 93.25% | 131 |
| Init10_24 |  | 1079985 | 77.58% | 1392031 | 93.81% | 128 |
| Init10_25 |  | 2265079 | 78.31% | 2892518 | 93.51% | 131 |
| Init10_2 |  | 1296420 | 77.83% | 1665709 | 93.49% | 129 |
| Init10_3 |  | 1552724 | 77.75% | 1997099 | 93.45% | 128 |
| Init10_4 |  | 849054 | 77.75% | 1092024 | 93.38% | 130 |
| Init10_5 |  | 2331808 | 77.22% | 3019682 | 93.27% | 128 |
| Init10_6 |  | 1003606 | 77.81% | 1289855 | 93.48% | 129 |
| Init10_7 |  | 792190 | 78.56% | 1008424 | 92.94% | 131 |
| Init10_8 |  | 412740 | 78.14% | 528209 | 92.22% | 129 |
| Init10_9 |  | 875707 | 77.83% | 1125090 | 92.60% | 130 |
| Init5_10 | none | 892973 | 68.83% | 1297329 | 92.91% | 129 |
| Init5_11 |  | 598928 | 66.44% | 901421 | 91.02% | 123 |
| Init5_12 |  | 877583 | 67.37% | 1302673 | 91.54% | 125 |
| Init5_13 |  | 543242 | 67.12% | 809411 | 91.23% | 126 |
| Init5_14 |  | 1128592 | 68.92% | 1637466 | 92.64% | 127 |
| Init5_15 |  | 755837 | 68.41% | 1104866 | 92.77% | 128 |
| Init5_16 |  | 1003246 | 67.92% | 1477078 | 91.87% | 126 |
| Init5_17 |  | 1352756 | 70.32% | 1923598 | 93.78% | 129 |
| Init5_18 |  | 1702741 | 70.46% | 2416618 | 93.82% | 129 |
| Init5_19 |  | 1089710 | 70.08% | 1554849 | 93.94% | 127 |
| Init5_1 |  | 1953959 | 69.77% | 2800412 | 93.50% | 128 |
| Init5_20 |  | 1767532 | 70.59% | 2504040 | 94.11% | 129 |
| Init5_21 |  | 1377724 | 70.52% | 1953658 | 94.04% | 129 |
| Init5_22 |  | 1892556 | 70.38% | 2689148 | 93.87% | 128 |
| Init5_23 |  | 1741974 | 70.60% | 2467357 | 93.82% | 130 |
| Init5_24 |  | 1121829 | 70.00% | 1602588 | 94.16% | 127 |
| Init5_25 |  | 1735333 | 70.59% | 2458367 | 93.95% | 130 |
| Init5_2 |  | 1371734 | 70.19% | 1954230 | 94.05% | 129 |
| Init5_3 |  | 1418713 | 69.65% | 2036908 | 93.50% | 127 |
| Init5_4 |  | 1105053 | 69.69% | 1585677 | 93.91% | 128 |
| Init5_5 |  | 2408286 | 69.73% | 3453939 | 93.63% | 128 |
| Init5_6 |  | 1109105 | 69.32% | 1600024 | 93.55% | 127 |
| Init5_7 |  | 1205427 | 68.24% | 1766548 | 92.57% | 127 |
| Init5_8 |  | 767200 | 66.57% | 1152441 | 90.83% | 123 |
| Init5_9 |  | 778029 | 67.28% | 1156483 | 91.58% | 123 |
| Init5_10 | m^6^A IP | 524476 | 76.59% | 684793 | 92.02% | 132 |
| Init5_11 |  | 429119 | 76.91% | 557920 | 92.05% | 135 |
| Init5_12 |  | 386936 | 77.19% | 501305 | 91.83% | 134 |
| Init5_13 |  | 195470 | 76.73% | 254738 | 91.75% | 134 |
| Init5_14 |  | 545466 | 77.04% | 708046 | 92.12% | 133 |
| Init5_15 |  | 447512 | 76.38% | 585932 | 92.35% | 131 |
| Init5_16 |  | 547671 | 76.31% | 717656 | 91.99% | 134 |
| Init5_17 |  | 1031570 | 76.79% | 1343287 | 92.82% | 133 |
| Init5_18 |  | 1352095 | 76.71% | 1762714 | 93.07% | 134 |
| Init5_19 |  | 451461 | 77.17% | 585023 | 93.13% | 133 |
| Init5_1 |  | 1211562 | 76.93% | 1574950 | 93.24% | 132 |
| Init5_20 |  | 917968 | 76.99% | 1192252 | 92.84% | 132 |
| Init5_21 |  | 994349 | 77.23% | 1287493 | 93.08% | 134 |
| Init5_22 |  | 918565 | 77.72% | 1181838 | 93.00% | 135 |
| Init5_23 |  | 968018 | 77.48% | 1249399 | 92.83% | 135 |
| Init5_24 |  | 588275 | 77.20% | 762062 | 93.48% | 132 |
| Init5_25 |  | 1071371 | 77.82% | 1376750 | 93.02% | 135 |
| Init5_2 |  | 603993 | 76.84% | 785993 | 92.96% | 132 |
| Init5_3 |  | 842096 | 76.30% | 1103675 | 92.90% | 130 |
| Init5_4 |  | 543962 | 77.13% | 705287 | 92.90% | 133 |
| Init5_5 |  | 1708691 | 75.83% | 2253458 | 92.80% | 131 |
| Init5_6 |  | 632821 | 76.88% | 823085 | 93.06% | 132 |
| Init5_7 |  | 656215 | 77.32% | 848744 | 92.51% | 135 |
| Init5_8 |  | 259271 | 76.80% | 337585 | 91.63% | 132 |
| Init5_9 |  | 414680 | 76.96% | 538835 | 92.34% | 132 |

**Table S4. Clinical baseline characteristics of the participants for SLIM-m^6^A-seq**

|  | Reference Range | T2D  (n = 10) | T2D+AMI  (n = 10) | *P* value |
| --- | --- | --- | --- | --- |
| Age, year | - | 67.2 ± 7.8 | 67.6 ± 9.3 | 0.918 |
| Sex, male% | - | 57.8 | 70.6 | 0.628 |
| SBP, mmHg | 90-139 | 129 ± 14 | 126 ± 25 | 0.752 |
| DBP, mmHg | 60-89 | 75 ± 12 | 79 ± 10 | 0.336 |
| Liver function |  |  |  |  |
| ALT, U/L | 9-50 | 18 (11-23) | 21（19-58） | 0.053 |
| GGT, U/L | 8-57 | 21 (16-42) | 22 (16-53) | 0.971 |
| ALP, U/L | 30-120 | 87 (80-120) | 80 (65-111) | 0.190 |
| TBIL, μmol L^-1^ | 5-21 | 13 (8-21) | 16 (11-22) | 0.579 |
| DBIL, μmol L^-1^ | 0-7 | 2.3 (1.6-4.6) | 3 (2-4) | 0.684 |
| IDBIL, μmol L^-1^ | 1.5-18 | 10.3 (6.4-15.6) | 13 (9-18) | 0.529 |
| TP, g/L | 65-85 | 69.3 ± 5.7 | 61.6 ± 4.9 | 0.005** |
| Albumin, g/L | 40-55 | 39.5 ± 5.2 | 35.9 ± 2.7 | 0.067 |
| Globulin, g/L | 20-40 | 30 ± 5 | 26 ± 4 | 0.058 |
| Cardiac biomarkers |  |  |  |  |
| AST, U/L | 15-40 | 23 (15-25) | 154 (69-446) | <0.001*** |
| CK, U/L | <171 | 88 (60-163) | 696 (185-1701) | 0.001** |
| CKMB, U/L | 0-25 | 17 (11-22) | 82 (26-239) | 0.001** |
| HBDH, U/L | 74-199 | 137 (120-150) | 396 (325-586) | 0.001** |
| LDH, U/L | 125-243 | 180 (165-193) | 423 (246-644) | <0.001*** |
| LDH1, U/L ^1^ | 15-65 | 29 (22-33) | 179 (117-336) | 0.001** |
| hsTnI, pg/mL | 0-26.2 | 9.3 (2.5-20.1) | 11018.8 (1830.8-25718.8) | 0.001** |
| BNP, pg/mL | <100 | 31 (25-58) | 210 (66-931) | 0.053 |
| Renal function |  |  |  |  |
| BUN, mmol/L | 2.8-7.6 | 5.5 (5.1-7.8) | 5.3 (4.5.-6.8) | 0.143 |
| Creatinine, μmol/L | 64-104 | 63.1 (56.3-81.3) | 81.8 (69.1-98.7) | 0.021* |
| UA, μmol/L | 208-428 | 402 (259-487) | 429 (359-480) | 0.541 |
| Glucose and lipids |  |  |  |  |
| Glucose, mmol/L | 3.9-6.1 | 8.5 (5.3-12.7) | 12.2 (6.4-18.9) | 0.368 |
| TC, mmol/L | <5.18 | 4.5 (3.6-5.1) | 4.1 (3.3-6.4) | 0.971 |
| Triglycerides, mmol/L | <1.7 | 1.2 (0.7-2.2) | 1.9 (1.0-2.5) | 0.247 |
| HDL-C, mmol/L | >1.04 | 1.1 (0.7-1.4) | 0.9 (0.7-1.1) | 0.481 |
| LDL-C, mmol/L | <3.37 | 2.6 (2.3-3.5) | 2.9 (1.9-4.2) | 0.631 |
| NEFA, umol/L | 129-769 | 458 (368-571) | 1108 (415-1524) | 0.065 |
| Inflammatory biomarkers |  |  |  |  |
| WBC, 10^9^/L | 3.5-9.5 | 5.3 (5.0-5.8) | 9.9 (6.6-14.4) | <0.001*** |
| Neutrophil, 10^9^/L | 1.8-6.3 | 3.2 (2.5-3.9) | 7.7 (4.2-13.1) | <0.001*** |
| Neutrophil, % | 40-75 | 55.0（51.1-64.3） | 74.9（65.7-82.3） | 0.007** |
| Lymphocyte, 10^9^/L | 1.1-3.2 | 1.8 (1.3-1.8) | 1.5 (1.1-1.8) | 0.400 |
| Lymphocyte, % | 20-50 | 27.7 ± 12.3 | 14.3 ± 8.3 | 0.010* |
| Monocyte, 10^9^/L | 0.1-0.6 | 0.43 ± 0.13 | 0.95 ± 0.69 | 0.041* |
| Monocyte, % | 3-10 | 7.8 ± 2.0 | 7.8 ± 2.6 | 0.947 |
| Eosinophils, 10^9^/L | 0.02-0.52 | 0.12 ± 0.07 | 0.12 ± 0.17 | 0.970 |
| Eosinophils, % | 0.4-8 | 2.2 ± 1.2 | 1.3 ± 1.6 | 0.172 |
| Basophils, 10^9^/L | 0-0.06 | 0.03 ± 0.01 | 0.04 ± 0.05 | 0.601 |
| Basophils, % | 0-1.0 | 0.5 ± 0.2 | 0.5 ± 0.3 | 0.611 |
| NLR | NA | 1.6 (1.4-3.0) | 4.3 (3.0-12.3) | 0.003** |
| IL6, pg/mL | 0-7 | NA | 25 (10-176) | NA |

Values are presented as mean ± SD or median (IQR). **P* < 0.05, ***P* < 0.01, ****P* < 0.001 compared to the control group. NA, not avaliable.

Abbreviations: SBP, systolic blood pressure; DBP, diastolic blood pressure; ALT, alanine transaminase; AST, aspartate transaminase; ALP, alkaline phosphatase; GGT, gamma-glutamyl transpeptidase; TBIL, total bilirubin; DBIL, direct bilirubin; IDBIL, indirect bilirubin; TP, total protein; BUN, blood urea nitrogen; CK, creatine kinase; UA, uric acid; TC, total cholesterol; HDL-C, high density lipoprotein cholesterol; LDL-C, low-density lipoprotein cholesterol; NEFA, free fatty acid; CKMB, creatine kinase-MB; HBDH, alpha-hydroxybutyric dehydrogenase; LDH, lactate dehydrogenase; LDH1, Lactate dehydrogenase isoenzyme 1; hsTnI, high-sensitivity cardiac troponin I; BNP, brain natriuretic peptide; WBC, white blood cell count; NLR, neutrophil to lymphocyte ratio; IL6, interleukin-6.

**Table S5. Differential Genes in gene expression and m^6^A modification explored by SLIM-m^6^A-seq**

| Gene | Function | Reference |
| --- | --- | --- |
| *CALML4* | calcium ion binding, which correlates with cardiac functions | *N Engl J Med.* 2008;358(18):1899-1908. |
| *PCDHGA10* |  | *Int J Cardiol.* 2021;344:149-159. |
| *SYT17* |  | *Circulation.* 2019;139(24):2778-2792. |
| *IL12RB2* | an anti-atherogenic function cytokine receptor subunit of IL-35 | *Int Immunopharmacol.* 2022;110: 108938.  *Circ Res.* 2019;124(9):1323-1336. |
| *THP1* | involved in synthesis of 5-methoxytryptophan (5-MTP) | *J Biomed Sci.* 2020;27(1):79. |
| *BSPRY* | involved in ubiquitination |  |
| *UCHL3* |  |  |
| *ZNF135* | contain zinc finger domain and function in regulation of transcription |  |
| *ZBTB12* |  |  |
| *ZNF217* |  |  |
| *TNFSFS14* | play a pathogenic role in atherogenesis and plaque destabilization in AMI | *Blood.* 2006;108(3):928-935. |
| *IL1B* | a proinflammatory cytokine produced by activated macrophages and monocytes | *Ann N Y Acad Sci.* 1998;856:1-11. |
| *TLR4* | contributes to vascular inflammation | Cardiovasc Res. 2020;116(1):226-236. |
| *TLR5* | deficiency exacerbates cardiac injury and inflammation induced by myocardial ischaemia-reperfusion in the mouse | *Clin Sci.* 2015;129(2):187-198. |
| *CCR2* | CCR2-induced inflammation and oxidative stress in the heart are involved in the development of diabetic cardiomyopathy | *Diabetes.* 2019;68(11):2063-2073. |
| *NLRP12* | Nlrp12 mutation causes C57BL/6J strain-specific defect in neutrophil recruitment | *Nat Commun.* 2016;7:13180. |
| *S100A12* | people died from sudden cardiac death has found high expression levels of S100A12 in coronary artery smooth muscle in the ruptured plaques, especially in diabetics | *Arterioscler Thromb Vasc Biol.* 2014;34(12):2695-9. |
| *CASP4* | Evolves in apoptosis | *Cell Death Dis.* 2012;3(6):e330. |
| *BCL6* | play a role in protecting mature cardiac myocytes from eosinophilic inflammation | *Cardiovasc Res.* 1999;42(3):670-9. |
| *TNFSF14* | Circulating TNFSF14 predicts clinical outcome in patients with stable coronary artery disease | *Arterioscler Thromb Vasc Biol.* 2019;39(6):1240-1252. |
| *CEACAM1* | angiogenesis activators | *Arterioscler Thromb Vasc Biol*. 2021; 41(1):186-199.  *J Am Coll Cardiol.* 2020; 76(14):1660-1670.  *Signal Transduct Target Ther.* 2022;7(1):78. |
| *TGFA* |  |  |

**Table S6. Clinical baseline characteristics of the participants for HPLC-MS/MS and RT-qPCR**

|  | reference Range | T2D  (n = 64) | T2D+AMI  (n = 51) | *P* value |
| --- | --- | --- | --- | --- |
| Age, year | - | 61.6±11.0 | 63.4±13.1 | 0.433 |
| Sex, male% | - | 57.8 | 70.6 | 0.157 |
| SBP, mmHg | 90-139 | 131± 17 | 128 ±20 | 0.395 |
| DBP, mmHg | 60-89 | 78 ± 21 | 78 ± 14 | 0.861 |
| Liver function |  |  |  |  |
| ALT, U/L | 9-50 | 20 (14-31) | 42.0 (21-62) | < 0.001*** |
| GGT, U/L | 8-57 | 27 (17-45) | 30 (21-54) | 0.149 |
| ALP, U/L | 30-120 | 89 (69-111) | 85 (70-106) | 0.840 |
| TBIL, μmol L^-1^ | 5-21 | 11 (8-15) | 14 (10-18) | 0.01* |
| DBIL, μmol L^-1^ | 0-7 | 2 (2-3) | 3 (2-4) | < 0.001*** |
| IDBIL, μmol L^-1^ | 1.5-18 | 9 (7-12) | 11 (8-14) | 0.034* |
| TP, g/L | 65-85 | 68 ± 7 | 63 ± 6 | 0.001** |
| Albumin, g/L | 40-55 | 41± 4 | 37 ± 6 | < 0.001*** |
| Globulin, g/L | 20-40 | 27 ± 4 | 27 ± 4 | 0.965 |
| Renal function |  |  |  |  |
| BUN, mmol/L | 2.8-7.6 | 5.4 (4.4-6.6) | 5.3 (4.5.-6.8) | 0.763 |
| Creatinine, μmol/L | 64-104 | 60.5 (49.2-74.9) | 74.9 (63.0-100.0) | < 0.001*** |
| UA, μmol/L | 208-428 | 337 (275-410) | 385 (292-491) | 0.06 |
| Glucose and lipids |  |  |  |  |
| Glucose, mmol/L | 3.9-6.1 | 10.7 (8.6-14.0) | 11.7 (7.2-14.7) | 0.757 |
| TC, mmol/L | < 5.18 | 4.94 (3.54-5.98) | 4.28 (3.72-5.36) | 0.185 |
| Triglycerides, mmol/L | < 1.7 | 1.63 (1.16-2.79) | 1.60 (1.14-2.13) | 0.707 |
| HDL-C, mmol/L | > 1.04 | 1.02 (0.85-1.23) | 0.82 (0.74-1.05) | < 0.001*** |
| LDL-C, mmol/L | < 3.37 | 2.94 (2.19-3.85) | 2.92 (2.41-3.33) | 0.621 |
| NEFA, umol/L | 129-769 | 654 (350-767) | 575 (407-812) | 0.440 |
| Cardiac biomarkers |  |  |  |  |
| AST, U/L | 15-40 | 19 (15-24) | 82 (29-248) | < 0.001*** |
| CK, U/L | < 171 | 76 (54-109) | 696 (185-1701) | < 0.001*** |
| CKMB, U/L | 0-25 | 15 (12-17) | 82 (26-239) | < 0.001*** |
| HBDH, U/L | 74-199 | 120 (103-135) | 396 (325-586) | < 0.001*** |
| LDH, U/L | 125-243 | 178 (145-208) | 423 (246-644) | < 0.001*** |
| LDH1, U/L ^1^ | 15-65 | 27 (22-31) | 179 (117-336) | < 0.001*** |
| hsTnI, pg/mL | 0-26.2 | 3.0 (1.8-4.9) | 11018.8 (1830.8-25718.8) | < 0.001*** |
| BNP, pg/mL | < 100 | 25 (17-40) | 210 (66-931) | < 0.001*** |
| Inflammatory biomarkers | |  |  |  |
| WBC, 10^9^/L | 3.5-9.5 | 6.2 (4.8-7.2) | 9.1 (6.5-11.1) | < 0.001*** |
| Neutrophil, 10^9^/L | 1.8-6.3 | 3.5 (2.5-4.4) | 7.0 (4.5-9.1) | < 0.001*** |
| Lymphocyte, 10^9^/L | 1.1-3.2 | 1.8 (1.4-2.2) | 1.5 (1.0-2.1) | 0.03* |
| NLR | NA | 1.7 (1.4-2.3) | 4.81 (2.38-7.80) | < 0.001*** |
| IL6, pg/mL | 0-7 | NA | 25 (13-42) | NA |

Values are presented as mean ± SD or median (IQR). **P* < 0.05, ***P* < 0.01, ****P* < 0.001 compared to the control group. NA, not avaliable.

Abbreviations: SBP, systolic blood pressure; DBP, diastolic blood pressure; ALT, alanine transaminase; AST, aspartate transaminase; ALP, alkaline phosphatase; GGT, gamma-glutamyl transpeptidase; TBIL, total bilirubin; DBIL, direct bilirubin; IDBIL, indirect bilirubin; TP, total protein; BUN, blood urea nitrogen; CK, creatine kinase; UA, uric acid; TC, total cholesterol; HDL-C, high density lipoprotein cholesterol; LDL-C, low-density lipoprotein cholesterol; NEFA, free fatty acid; CKMB, creatine kinase-MB; HBDH, alpha-hydroxybutyric dehydrogenase; LDH, lactate dehydrogenase; LDH1, Lactate dehydrogenase isoenzyme 1; hsTnI, high-sensitivity cardiac troponin I; BNP, brain natriuretic peptide; WBC, white blood cell count; NLR, neutrophil to lymphocyte ratio; IL6, interleukin-6.

**Table S7. Primers used for RT-qPCR**

| Name | Sequence (from 5’ to 3’) |
| --- | --- |
| *FTO* forward primer | CTTCACCAAGGAGACTGCTATTTC |
| *FTO* reverse primer | CAAGGTTCCTGTTGAGCACTCTG |
| *GAPDH* forward primer | ATGACATCAAGAAGGTGGTG |
| *GAPDH* forward primer | CATACCAGGAAATGAGCTTG |
